# Proteasome 19S RP Binding to the Sec61 Channel Plays a Key Role in ERAD

**DOI:** 10.1371/journal.pone.0117260

**Published:** 2015-02-06

**Authors:** Marie-Luise Kaiser, Karin Römisch

**Affiliations:** Department of Microbiology, Faculty of Natural Sciences and Technology VIII, Saarland University, 66123, Saarbrücken, Germany; Cambridge University, UNITED KINGDOM

## Abstract

Import of secretory proteins into the Endoplasmic Reticulum (ER) is an established function of the Sec61 channel. The contribution of the Sec61 channel to export of misfolded proteins from the ER for degradation by proteasomes is still controversial, but the proteasome 19S regulatory particle (RP) is necessary and sufficient for extraction of specific misfolded proteins from the ER, and binds directly to the Sec61 channel. In this work we have identified an import-competent *sec61* mutant, S353C, carrying a point mutation in ER-lumenal loop 7 which reduces affinity of the cytoplasmic face of the Sec61 channel for the 19S RP. This indicates that the interaction between the 19S RP and the Sec61 channel is dependent on conformational changes in Sec61p hinging on loop 7. The *sec61-S353C* mutant had no measurable ER import defects and did not cause ER stress in intact cells, but reduced ER-export of a 19S RP-dependent misfolded protein when proteasomes were limiting in a cell-free assay. Our data suggest that the interaction between the 19S RP and the Sec61 channel is essential for the export of specific substrates from the ER to the cytosol for proteasomal degradation.

## Introduction

Proteins destined for secretion enter the secretory pathway by translocation through the Sec61 channel in the membrane of the endoplasmic reticulum (ER) [1]. Secretory and membrane proteins which fail to fold in the ER are not allowed to progress through the secretory pathway, but are returned from the ER to the cytosol for degradation by proteasomes (ER-associated degradation, ERAD) [1, 2]. The composition of the retrotranslocation channel is controversial, but retrograde transport of many soluble and some transmembrane substrates is dependent on the Sec61 channel [1, 2]. Part of the controversy stems from the fact that most *sec61* mutants initially characterized for ERAD had defects in transport across the ER membrane in both directions, which made it difficult to differentiate direct effects on ERAD-related export from indirect effects caused by altered import of ER-resident proteins required for ERAD [3, 4, 5].

The Sec61 channel consists of three proteins, Sec61p, Sbh1p, and Sss1p in yeast, equivalent to Sec61 α, β, γ in mammals [6]. This channel on its own mediates cotranslational protein import into the ER, during which the ribosome binds to Sec61p and Sbh1p [7, 8]. A mutation in cytosolic loop 8 of Sec61p reduces its affinity for ribosomes [9]. The crystal structure of a homologous archaeal channel in the closed conformation consists of a single Sec61 α/β/γ heterotrimer [10]. When comparing it to the structure of the Sec61 channel during initiation of ER import it is evident that concomitant with the separation of transmembrane domains 2 and 7 by signal peptide insertion into the lateral gate a large motion around ER-lumenal loop 7 (L7) of Sec61p takes place [11, 12]. Thus during Sec61 channel opening for protein import into the ER a conformational change initiated at the cytosolic face of the channel is transmitted across the membrane and affects channel structure on the lumenal side.

The first indication that mobility around L7 of Sec61p might be important for channel function was the identification of amino acid alterations in L7 of Antarctic and Arctic fishes that were not present in L7 of mesophile fish species [13]. Cold-adaptation of enzymes frequently involves amino acid substitutions that increase flexibility and mobility of proteins to allow conformational changes at low temperature, hence we proposed that substitutions in L7 of polar fishes improved Sec61 channel function in the cold [13]. One of the positions substituted in L7 of polar fishes (Y344H) was later found mutated in a screen for mutant mice prone to diabetes [14]. Pancreatic dysfunction in the mutant mice likely develops due to accumulation of misfolded proteins in the ER of pancreatic beta cells [14]. Mutation of the homologous position in yeast *SEC61* (Y345H) causes no defects in ER protein import, but a delay in export and ERAD of a soluble misfolded protein [15, 16]. A delay in soluble protein import into the ER was observed after insertion mutagenesis of L7 of yeast Sec61p [17]. Effects of these insertions in L7 on ERAD were not investigated, but a complete deletion of L7 in yeast Sec61p leads to profound defects in soluble protein transport through the channel in both directions, suggesting a role of L7 in transverse opening of the Sec61 channel for import or export [16].

The 26S proteasome is formed by two subparticles, the 20S proteolytic core particle (CP) and the 19S regulatory particle (RP), which contains ubiquitin-binding proteins, deubiquitating enzymes, 6 AAA-ATPases, and a number of proteins of unknown function [18]. At the end of proteasomal substrate turnover, the 19S and 20S particles dissociate from each other in a process dependent on ATP-hydrolysis, and the 19S RP dissociates further into its base and lid subparticles [19]. This assembly/disassembly cycle of the proteasome may allow for exchange of the 19S lid with the structurally homologous COP9 signalosome [20]. Individual proteasome 19S subparticles can also function on their own in transcription [21].

The pathway and the mechanism by which ERAD substrates are extracted from the ER membrane is still under debate. The Sec61 channel, the multispanning transmembrane protein Der1p, and the E3 ligase Hrd1p all have been suggested as retrograde protein transport channel [1, 2]. The *DER1* gene, however, can be deleted in yeast with little or no effect on ERAD of many substrates, but due to its proximity to specific ERAD substrates during export is likely to be an accessory factor that helps these substrates to exit the ER [1, 2]. While ubiquitination by the Hrd1 ubiquitin ligase is critical for ERAD of many soluble and some transmembrane substrates, a chimaera composed of the Hrd1p enzymatically active RING domain fused directly to the the transmembrane domains of HMG-CoA reductase autoubiquitinates and is exported from ER membranes independendently of Hrd1p, suggesting that Hrd1p is not the export channel for this transmembrane ERAD substrate [22]. Attempts to reconstitute ERAD of the soluble Hrd1 substrate CPY* with proteoliposomes containing purified Hrd1p failed, making it unlikely that Hrd1p on its own can transport soluble proteins from the ER lumen to the cytosol despite its proximity to this soluble substrate at the late stages of its membrane extraction [23].

For many substrates, the AAA-ATPase Cdc48p (p97 in mammals) is critically involved in export from the ER [2], but the 19S RP on its own can also promote misfolded protein exit from both yeast and mammalian ER [24, 25]. The 19S RP can also cooperate with Cdc48p both during proteasomal degradation of substrates that are difficult to unfold and during extraction of proteins from the ER membrane for ERAD [26, 27]. In a cell-free system, proteasomes in the presence of ATP promote export and degradation of a soluble degradation substrate from ER to the cytosol [24, 25]. Export and degradation of this substrate can be uncoupled, and the 19S RP on its own is sufficient for export in this system [24]. Proteasomes bind to the Sec61 channel in yeast and mammalian ER, and compete with ribosomes for channel binding [28]. The interaction is ATP-dependent, and mediated by the 19S RP and protease-sensitive cytosolic loops of the Sec61 complex [28]. Binding is mediated by the AAA-ATPase ring of the base of the 19S RP [29]. The AAA-ATPase Cdc48p also binds to the Sec61 channel [28]. The Sec61p domain required for 19S RP binding to the Sec61 channel has not been defined yet, but it differs from the ribosome binding site [29]. A *sec61* mutant in which the lateral gate of Sec61p is partially open shows enhanced 19S RP binding suggesting that a specific conformation of the channel is required for the interaction [16].

**Fig 1:**
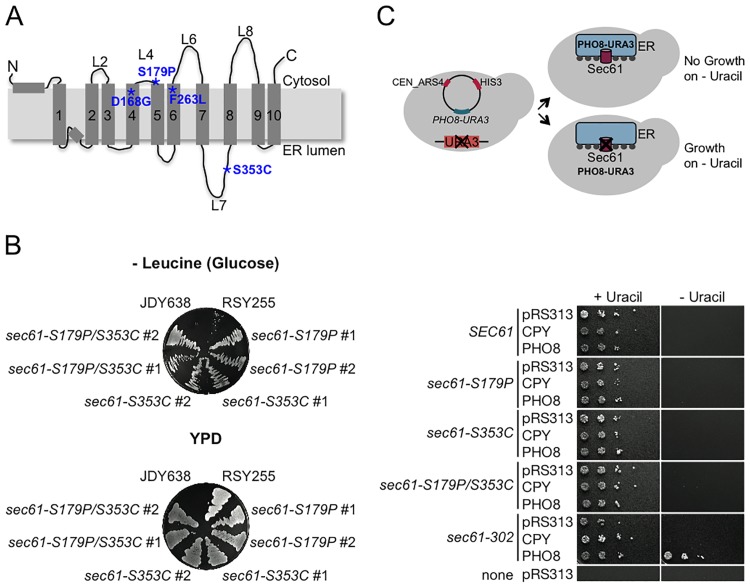
Individual point mutants derived from *sec61–302* are translocation competent. A: Topology model of Sec61p. The positions of the four point mutations in *sec61–302* are highlighted in blue with asterisks. B: Growth of isogenic wildtype JDY638 and *sec61* mutants on SC without LEU (Glucose) and YPD, respectively. RSY255 was used as wildtype control for *SEC61* under its own promoter; mutant growth was examined with two clones each (#1/#2). C: Effects of *sec61* mutations on protein import into the ER. Yeast were transformed with reporter plasmids pDN106 (pRS313-CPY-URA3; posttranslational) or pYN203 (pRS313-PHO8-URA3; cotranslational) or empty vector pRS313 (control). Transformants were grown on SC with HIS/LEU with or without URA. Cells deficient for ER protein import grow on medium without uracil whereas cells proficient for ER import do not.

We have previously characterized a *sec61* mutant containing four point mutations, *sec61–302*, with reduced affinity for the 19S RP [29] (Fig. 1). In this paper, we identify the point mutation in *sec61–302* which is responsible for the defect in proteasome binding (*sec61-S353C*). Proteasomes bind to the cytosolic face of the Sec61 channel, but the mutation is located in ER-lumenal L7 of Sec61p (Fig. 1) suggesting that S353C causes or prevents a conformational change in Sec61p that is transmitted to the cytosolic side and critical for 19S RP binding. We show that *sec61-S353C* mutant yeast are ER-import competent and that ER stress is not induced in the mutant which is consistent with its modest effects on ERAD in intact cells. In a cell-free ERAD assay based on yeast ER membranes in which proteasomes are limiting, however, the *sec61-S3553C* mutant displayed a striking delay in ERAD of a 19S RP-dependent substrate. Our data suggest that the 19S RP interaction with the Sec61 channel is governed by conformational changes hinging on ER-lumenal L7, and that the Sec61 channel/19S RP interaction is instrumental in the export of a subset of misfolded proteins from the ER during ERAD.

## Materials and Methods

*S*. *cerevisiae* strains used in this study are listed in Table 1, plasmids in Table 2, primers in Table 3.

**Table 1:** *S*. *cerevisae* strains used in this study.

Name	Genotype	Reference
GPY60/ KRY40	*MATα leu2–3,112 ura3–52 his4–579 trp1–289 prb1 pep4::URA3 gal2*	[44]
KRY47	*MATα leu2–3,112 ura3–52*	[3]
KRY161	*MATα ade2–1 ura3–1 his3–11,15 leu2–3,112 trp1–1 can1–100 prc1–1*	[47]
KRY200	*MATα can1–100 leu2–3,112 his3–11,15 trp1–1 ura3–1 ade2–1sec61::HIS3 pDQ1[sec61–32]*	[4]
KRY201	*MATα can1–100 leu2–3,112 his3–11,15 trp1–1 ura3–1 ade2–1 sec61::HIS3 pDQ[sec61–41]*	[4]
KRY221	*MATα sec61–3 trp1–1 leu2–3,112 ura3–52*	[55]
KRY275	*leu2–3,112 ura3 his3–11,15*	[57]
KRY333	*his3Δ200 leu2–3,112 lys2–801 trp1Δ63 ura3–52 RPT1^FH^::YIplac21(URA3)*	[34]
KRY461	*MATα sec61::HIS3 leu2 trp1 prc1–1 his3 ura3 [pGAL-SEC61-URA3]*	our lab, unpublished
KRY706	BMA38a, *kanr-pGAL-SEC61*, *his2-Δ200 leu2–3*,*112 trp1-Δ1 ura3–1 ade2–1 can1–100* [pCEN*-LEU2-SEC61*]	[29]
KRY712	BMA38a, *kanr-pGAL-SEC61*, *his2-Δ200 leu2–3*,*112 trp1-Δ1 ura3–1 ade2–1 can1–100* [pCEN*-LEU2-sec61–302*]	[29]
KRY715	BMA38a, *kanr-pGAL-SEC61*, *his2-Δ200 leu2–3*,*112 trp1-Δ1 ura3–1 ade2–1 can1–100* [pCEN*-LEU2-sec61–303*]	[29]
KRY849	BMA38a, *kanr-pGAL-SEC61*, *his2-Δ200 leu2–3*,*112 trp1-Δ1 ura3–1 ade2–1 can1–100* [pCEN*-LEU2-sec61-S179P*]	This study
KRY850	BMA38a, *kanr-pGAL-SEC61*, *his2-Δ200 leu2–3*,*112 trp1-Δ1 ura3–1 ade2–1 can1–100* [pCEN*-LEU2-sec61-S353C*]	This study
KRY851	BMA38a, *kanr-pGAL-SEC61*, *his2-Δ200 leu2–3*,*112 trp1-Δ1 ura3–1 ade2–1 can1–100* [pCEN*-LEU2-sec61-S179P/S353C*]	This study
KRY852	*MATα leu2–3*,*112 ura3–52* [pRS306*-truncsec61-S179P*]	This study
KRY853	*MATα leu2–3*,*112 ura3–52* [pRS306*-truncsec61-S353C*]	This study
KRY854	*MATα leu2–3*,*112 ura3–52* [pRS306*-truncsec61-S179P/S353C*]	This study
JDY638/ KRY858	BMA38a, *kanr-pGAL-SEC61*, *his3-Δ200 leu2–3*,*112 trp1-Δ1 ura3–1 ade2–1 can1–100*	[29]
KRY879	*MATα ade2–1 ura3–1 his3–11,15 leu2–3,112 trp1–1 can1–100 der1::natNT2*	[54]
KRY880	*MATα ade2–1 ura3–1 his3–11,15 leu2–3,112 trp1–1 can1–100 prc1–1 der1::natNT2*	[54]

**Table 2:** Plasmids.

Plasmid	Characteristics	Reference
p416pΔgpαf	overexpression of pΔgpαf (*URA3*), contains: MET25 promoter	[58]
pαF3Q	gene for pΔgpαf in MC1600, linearization with *Sal*I	[59]
pBW11	WT *SEC61* in pRS315	[32, 55]
pDN106	expression of CPYp-URA3p fusion protein (pRS313-*CPY-URA3*; *HIS*)	[29]
pDN431	CPY*-HA in YCP50 (*URA3*)	[60]
pGEM2αF	gene for ppαf (WT; serine variant) in pGEM; SP6 promoter; linearization with *Sal*I	[59]
pJC30	*UPRE-LacZ* reporter construct in pRS314	[61]
pJC31	CYC1 TATA box fused to LacZ in pRS314	[61]
pJEY203	PHO8p-Ura3p fusion protein (pRS313-*PHO8-URA3*; *HIS*)	[29]
pSM101	KWW-HA (*URA3*)	[49]

**Table 3:** Primers.

Name	Sequence (5’→3’)	Length (bp)	Application
5’ *Hin*dIII SEC61 5’UTR #-445	AAGCTTGCTATAAGCTAGAATGTATTGAATGTATTC	36	SOE-PCR full length *sec61* mutants
5’ *Eco*RI SEC61 #57	GAATTCAGTGATTGCTCCAGAAAGGAAGGTTCC	27	truncation of *sec61–302*, *sec61–303*
5’ *Hind*III SEC61 #57	AAGCTTAGTGATTGCTCCAGAAAGGAAGGTTCC	27	SOE-PCR truncated *sec61* mutants
5’ SOE SEC61 T201G	CTGTACTGGCTACGGGCCATGCTGGC	26	SOE-PCR truncated *sec61* mutants
3’ SOE SEC61 T201G	GCCAGCATGGCCCGTAGCCAGTACAG	26	SOE-PCR truncated *sec61* mutants
5’ SOE SEC61 T535C	GTTACGGCTTGGGTCCCGGTATTTCTCTG	29	SOE-PCR *sec61-S179P*, *sec61-S179P/S353C* (T535C)
3’ SOE SEC61 T535C	CAGAGAAATACCGGGACCCAAGCCGTAA	29	SOE-PCR *sec61-S179P*, *sec61-S179P/S353C* (T535C)
5’ SOE SEC61 C1058G	CATTAATGTCTTTATGCGAAGCTCTTCTGGAC	32	SOE-PCR *sec61-S353C*, *sec61-S179P/S353C* (C10598G)
3’ SOE SEC61 C1058G	GTCCAGAAGAGCTTCGCATAAAGACATTAATG	32	SOE-PCR *sec61-S353C*, *sec61-S179P/S353C* (C10598G)
3’ SEC61 3’UTR #1765 *Hin*dIII	AAGCTTGCGCATTTGCTTAAGCAAGGATACC	25	SOE-PCR *sec61* mutants
3’ SEC61 3’UTR #1765 *Xho*I	CTCGAGGCGCATTTGCTTAAGCAAGGATACC	25	SOE-PCR truncated *sec61–302*, *sec61–303*
5’ SEC61 CHR #403	GCAAGTAGAAAAACTGACACTGGTTCACG	29	verification of s*ec61* integration into *S*. *cerevisiae* genome
3’ pRS306 URA3 #621	GTTGACCCAATGCGTCTCCCTTGTC	25	verification of s*ec61* integration into *S*. *cerevisiae* genome
5’ *Sal*I YDJ1	GTCGACATGGTTAAAGAAACTAAGTTTTACGATATTCTAGG	35	control PCR for *sec61* integration
3’ YDJ1 *Xba*I	TCTAGATCATTGAGATGCACATTGAACACCTTC	27	control PCR for *sec61* integration
T3	ATTAACCCTCACTAAAGGGA	20	sequencing
T7	TAATACGACTCACTATAGGG	20	sequencing
SEC61 pBW11 SEQ	AAATAGAGGGAGGGGTGTGG	20	sequencing

### Mutant construction

Generation of the desired *sec61* mutants was by site-directed mutagenesis using PCR-driven splice overlap extension (SOE) [30]. The SOE-PCR protocol was as previously described [31]. *S*. *cerevisiae SEC61*, amplified from pBW11 (*LEU*, *CEN*, *SEC61*; ref. Table 2), was used as template [32]. During SOE-PCR fragments of the target sequence (*SEC61*) were amplified using gene-specific sets of mutagenic primers (Table 3). Mutagenic primers (Table 3) were designed complementary to each other containing the desired nucleotide exchange in the centre of the primer. Flanking primers, used for the extension of the final PCR product, contained *Hin*dIII (or *Eco*RI/*Xho*I for *sec61–302* and *sec61–302*) restriction sites (Table 3). The resulting PCR products were cloned into pRS315 (*CEN*, *LEU*; full-length *sec61*) and pRS306 (*URA3*; truncated *sec61*) to create the respective constructs. Integration of *sec61* mutants into the genome of the *S*. *cerevisiae* strain RSY255 (Table 1) at the correct chromosomal locus was verified by PCR on chromosomal DNA with the primers 5’ *Hin*dIII *SEC61* 5’UTR #-445 and 3’ pRS306 *URA3* #621 (Table 3). Transformants (pCEN-LEU2-sec61*) in the JDY638 (pGAL-*SEC61*) background were selected on SC medium containing 2% (w/v) galactose and 0.2% (w/v) raffinose and lacking leucine. Counterselection was on YPD and SC medium containing 2% glucose and lacking leucine. sec61*: *sec61-S179P*, *sec61-S353C*, *sec61-S179P/S353C*.

### Growth of *S*. *cerevisiae*

*S*. *cerevisiae* cells were grown at 30°C in YPD or in SC medium with continuous shaking at 225 rpm or on YPD or on drop-out plates at 30°C if not stated otherwise. To test tunicamycin (Tm) sensitivity, serial dilutions were prepared and 5 µl of each dilution containing 10^5^–10 cells were dropped onto YPD (+/-0.25 µg/ml Tm, 0.5 µg/ml Tm) plates or SC medium plates (+/-0.25 µg/ml Tm, 0.5 µg/ml Tm). Plates were incubated at indicated temperatures for 3 days.

### Translocation Reporter Assay

Newly generated *sec61* mutants (*sec61-S170P*, *sec61-S353C*, *sec61-S179P/S353C*) were transformed with reporter plasmids pDN106 (pRS313-*CPY-URA3*; posttranslational import; [29]), or pJN203 (pRS313-*PHO8-URA3*; cotranslational import; [29]), or the empty vector pRS313 (control). Transformants were selected on SC without HIS/LEU. Overnight cultures of positive transformants were prepared and serial dilutions of each strain were grown on SC without HIS/LEU/URA to monitor translocation defects or without HIS/LEU to monitor growth.

### Western Blot Analysis

Protein gel electrophoresis was routinely conducted using NuPAGE Novex Pre-Cast Bis-Tris gels (generally 4–12% gels, 1.5 mm, 10 wells) and the XCell SureLock Mini-Cell (both Invitrogen) if not stated otherwise. Protein detection was after transfer to nitrocellulose with appropriate antibodies and SuperSignal West Dura Extended Duration Chemiluminescent Substrate (Pierce) according to the supplier’s instructions. Signals were detected using the Molecular Imager ChemiDoc XRS System (BioRad; CCD camera detection) evaluated/quantified with the ImageLab software (BioRad). Rabbit polyclonal sera against prepro alpha factor, CPY, Sec61p N-terminus & Sec61p C-terminus had been raised in our lab, and were used at 1:2000; anti-FLAG M2 monoclonal mouse (Sigma) and polyclonal rabbit (Sigma) were used at 1:2000; polyclonal rabbit anti-HA (Sigma) at 1:5000; goat anti-rabbit HRP (Rockland) as secondary antibody 1:20,000 using chemiluminescence reagents (Pierce).

### Yeast Rough Microsomes and PK-RM

The isolation of rough microsomal membranes *from S*. *cerevisiae* was according to [3] and the samples aliquoted (50 µl), snap-frozen in liquid nitrogen and stored at -80°C. Preparation of ribosome- and proteasome-stripped membranes (PK-RM) from RM by treatment with puromycin and potassium acetate was modified from [33]. Stripped membranes were aliquoted into (25 µl), snap-frozen in liquid nitrogen and stored at -80°C. The concentration of PK-RMs was determined by Western Blotting against Sec61p using RMs as standard.

### Purification of *S*. *cerevisiae* Proteasome 26S Holoenzyme and 19 S RP subcomplex

The purification of *S*. *cerevisiae* 26 S proteasome and 19S RP (Regulatory Particle) was according to [24, 34, 35] using the yeast strain KRY333 which expresses a FLAG-tagged version of the Rpt1 19S RP subunit. Proteasome activity was monitored using the fluorogenic peptide substrate N-Succinyl-Leu-Leu-Val-Tyr 7-Amido-4-Methylcoumarin (Suc-LLVY-AMC, Sigma). The assay was performed as described in [35].

### Reconstitution of Proteoliposomes & Proteasome Binding Assay

Reconstituted proteoliposomes were prepared according to [7, 36, 37] and reconstituted membranes were quantified by Western Blot against Sec61p using RMs as standard (0.25 eq, 0.5 eq, 0.75 eq, 1.0 eq, 1.5 eq of RMs). The proteasome binding assay was performed as described by [28]. In brief, 20 eq of reconstituted proteoliposomes were mixed with 2 pmole of 19S RP or 26S holoenzyme in 30 µl Binding Buffer (20 mM Hepes-KOH pH 7.2, 250 mM sucrose, 120 mM KOAc, 5 mM Mg(OAc)_2_, 5 mM ATP, 1 mM DTT). The reaction mix was first incubated on ice for 20 min followed by 10 min at RT. Next, 270 µl of Sucrose Cushion (20 mM Hepes-KOH pH 7.2, 2.5 M Sucrose, 120 mM NH_4_OAc, 5 mM Mg(OAc)_2_, 5 mM ATP, 1 mM DTT) were added and the sample was vortexed for 10 sec. 800 µl of Separating Cushion (20 mM Hepes-KOH pH 7.2, 1.8 M sucrose, 120 mM NH_4_OAc, 5 mM Mg(OAC)_2_, 5 mM ATP, 1 mM DTT) were added to a polycarbonate thickwall tube. The separating cushion was carefully underlaid with the sample (300 µl). The sample was topped off with 200 µl of binding buffer and centrifuged at 55,000 rpm, 4°C for 1 hr (TLS55, Optima MAX-XP Benchtop Ultracentrifuge). After ultracentrifugation, the sample was divided into nine fractions (from top to bottom). Individual fractions were TCA-precipitated, proteins resolved by SDS-PAGE, and proteasomes detected using anti-FLAG M2 antibody (1:2000, Sigma).

### Quantitative liquid ß-Galactosidase Assay

The quantitative liquid ß-Galactosidase assay was essentially as described by [38, 39]. All yeast strains of interest were transformed with the plasmids pJC30 (UPRE-LacZ reporter construct) and pJC31 (LacZ without UPRE, control) [40]. All cultures were prepared in duplicates. One culture was used as a positive control: when an OD_600_ of about 0.4 was reached, tunicamycin (Tm) was added to a final concentration of 1 µg/ml, incubated for 1h at 30°C, 220 rpm and ß-galactosidase assayed.

### Pulse-Chase Experiments

Pulse chase experiments were performed as described by [34, 41].

### *In Vitro* Transcription, Translation, and Retrotranslocation

Concentrated yeast cytosol was prepared using the yeast strain KRY275 according to [42, 43]. Yeast translation extract was prepared essentially as in [44]. The preparation was performed under RNase-free conditions. Transcripts were produced from linearized pDJ100 encoding pαF3Q. Protein was in vitro translated in the presence of [^35^S]-methionine, snap-frozen in liquid nitrogen in 50 µl aliquots, and stored at—80°C. The retrotranslocation substrate pΔgpαf imported into wild-type of mutant yeast microsomes and retrotranslocation time courses performed as described in [3], with varying concentrations of cytosol as indicated.

## Results

### Individual point mutants derived from *sec61–302* are translocation competent

Proteasomes bind to the cytosolic face of the Sec61 channel via the 19S RP, but the physiological relevance of this interaction is still unknown [28, 29]. We have previously identified a *sec61* mutant shown in Fig. 1A, *sec61–302*, with four point mutations which resulted in a cotranslational import defect and reduced affinity of the mutant Sec61 channel for proteasomes [29]. Two of the four point mutations (D168G, F263L) were also present in *sec61–303* which was also defective in cotranslational import into the ER, but did not have a proteasome binding defect [29]. In order to identify which of the remaining point mutations in *sec61–302* (S179P or S353C) was responsible for the proteasome binding defect, we generated *sec61* mutants carrying one or both mutations by splice-overlap extension PCR [40, 31]. The resulting *sec61* mutants were viable (Fig. 1 B). To analyze whether our new *sec61* mutants were defective in biosynthetic protein import into the ER, we transformed the strains with reporter plasmids encoding a fusion of the carboxypeptidase Y (CPY) signal peptide to the *URA3* gene, or a fusion of the Pho8p signal anchor to the *URA3* gene. In the yeast strains used the chromosomal copy of the *URA3* gene was deleted, thus the cells were only able to grow in the absence of uracil if the fusion protein remained in the cytosol due to a protein import defect into the ER (Fig. 1C, top) [29]. In this assay the *CPY*-*URA3* fusion monitors posttranslational import defects, whereas *PHO8-URA3* detects cotranslational import defects [45, 29]. As shown in Fig. 1C we confirmed the cotranslational import defect in the original *sec61–302* mutant by growth in the absence of uracil of the *PHO8-URA3* transformants, but neither *sec61-S179P*, nor *sec61-S353C*, nor the *sec61-S179P/S353C* double mutant had any detectable defects in co- or posttranslational import and hence did not grow on SC without uracil regardless of which fusion protein they expressed (Fig. 1C). Our data suggest that the cotranslational import defect in *sec61–302* is caused by the point mutations that it shares with *sec61–303* (D168G, F263; Fig. 1A) [29], and that S179P and S353C do not affect transport into the ER through the Sec61 channel.

### A mutation in ER-lumenal L7 of Sec61p, S353C, reduces affinity of the Sec61 channel for the 19S RP

**Fig 2:**
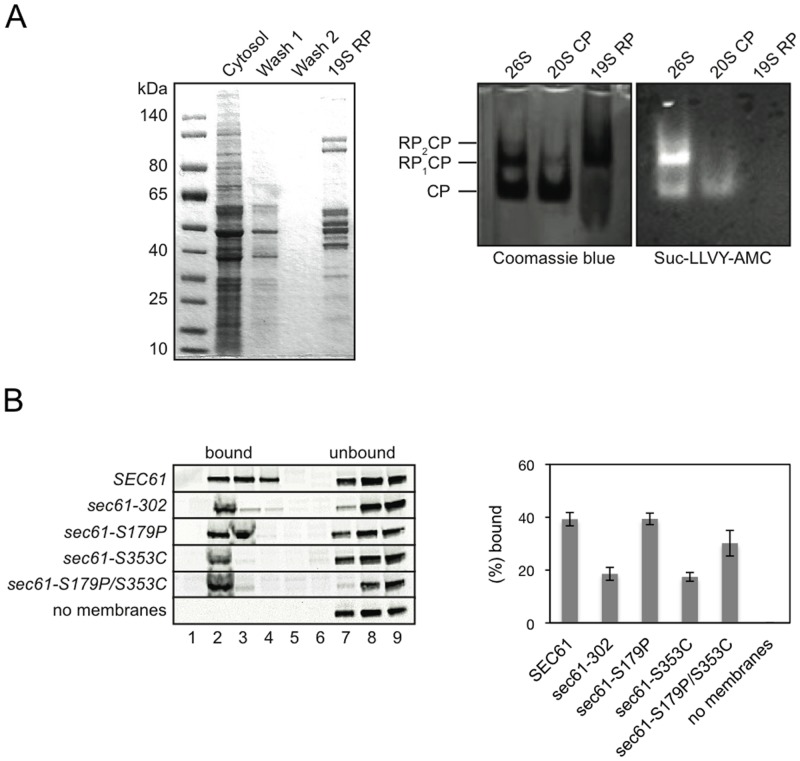
A mutation in ER-lumenal L7 of Sec61p, *sec61-S353C*, reduces affinity for the 19S RP. A: Left: Coomassie Blue-stained gel of purified FLAG-tagged 19S RP. Fractions from each step of purification were analyzed by 4–12% SDS-PAGE. Right: Verification of 19S RP purity. Purified 19S RP and, as controls, 20S CP and 26S proteasomes were resolved by native SDS-PAGE, and the gel incubated with the fluorogenic proteolysis substrate Suc-LLVY-AMC. B: Left: Effect of *sec61* mutations on 19S RP binding to Sec61 channels. Wild-type and *sec61* mutant proteoliposomes were incubated with 2 pmol purified 19S RP, membrane-bound and unbound 19S RP were separated by flotation in a sucrose gradient, and gradients divided into 9 fractions from the top. Proteins in each fraction were TCA-precipitated and resolved by SDS-PAGE; 19S RP was detected via Western Blotting using polyclonal anti-FLAG M2 rabbit antibody. Right: Quantitation of proteasome binding to *SEC61* wildtype and mutant channels. The experiment was performed 3 times; bars indicate standard error.

Since we had found a proteasome binding defect in *sec61–302* derived membranes, but not in membranes from *sec61–303* yeast, the reduced proteasome binding to *sec61–302* membranes must have been due to the mutations unique to this mutant (S179P, S353C; Fig. 1A) [29]. To identify the point mutation responsible for reduced 19S RP binding to the mutant Sec61 channel, we performed 19S RP binding assays with proteoliposomes derived from the mutants unique to *sec61–302*, S179P and S353C. We isolated FLAG-tagged 19S RP from liquid nitrogen-lysed cells by affinity chromatography [34] (Fig. 2 A, left). To demonstrate purity of the isolated 19S RP, we incubated a native gel containing proteolytically active 26S holo-proteasomes, proteolytically active 20S CP, and purified FLAG-tagged RP with the fluorogenic proteasome substrate Suc-LLVY-AMC [24]. Whereas 26S proteasomes and 20S CP were proteolytically active as expected, the 19S RP sample showed no fluorescence and hence no proteolytic activity indicating absence of contamination with 26S proteasomes (Fig. 2A, right).

We next incubated purified 19S RP with wildtype and *sec61* mutant proteoliposomes in the presence of ATP to allow binding to the Sec61 channel [28]. Membrane-bound and unbound 19S RP were separated by flotation in sucrose gradients and gradients divided into 9 fractions from top to bottom. Proteins in each fraction were precipitated with trichloroacetic acid and separated by SDS-PAGE. We detected the position of 19S RP in the gradient by Western Blotting using a polyclonal anti-FLAG M2 antibody (Fig. 2 B, left). Membrane-bound 19S RP migrated to the upper fractions (2–4) of the gradient, whereas unbound 19S RP remained in the lower fractions (7–9) (Fig. 2B, left). A sample without membranes was used as a control (Fig. 2B, left, bottom). As shown in the graph (Fig. 2B, right) under the conditions used in our experiment, 40% of 19S RP bound to proteoliposomes containing wild-type Sec61p, whereas only 20% of 19S RP bound to *sec61–302* membranes (Fig. 2B, right). This was due to reduced affinity of the 19S RP to the mutant Sec61 channels, as wildtype and mutant proteoliposomes contained equal amounts of Sec61p (not shown). Binding to *sec61-S353C* membranes was comparable to binding to *sec61–302* proteoliposomes, whereas binding to *sec61-S179P* membranes was comparable to wildtype membranes (Fig. 2B, right). The *sec61-S179P/353C* double mutant displayed an intermediate phenotype with respect to 19S RP binding (Fig. 2B, right). We conclude that the *sec61-S353C* mutation is responsible for the reduction of 19S RP binding to *sec61–302* membranes.

### The *sec61-S353C* mutation does not cause ER stress

**Fig 3:**
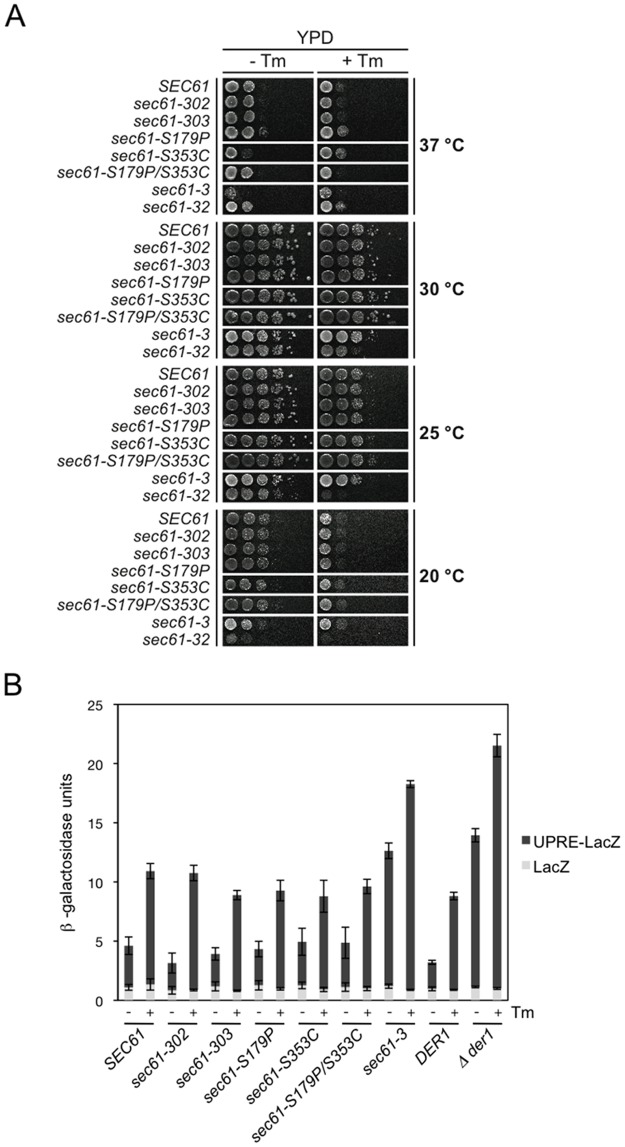
The *sec61-S353C* mutation does not cause ER stress. A: Growth of *sec61* mutants and *SEC61* wildtype on YPD at different temperatures was monitored +/- tunicamycin (0.5 µg/ml). Plates were incubated for 3 days. B: Liquid ß-galactosidase assay with *sec61* mutants and controls. Strains were transformed with plasmids pJC30 (UPRE-LacZ) or pJC31 (LacZ control) and ß-galactosidase activity monitored as described in Methods.

The results shown in Fig. 2 indicated reduced affinity of Sec61-S353C channels for the 19S RP. If the Sec61/19S RP interaction were important for export of misfolded proteins for ERAD, one would expect accumulation of misfolded proteins in the ER of the S353C mutant which would elicit ER stress resulting in growth defects, sensitivity to tunicamycin, or induction of the Unfolded Protein Response (UPR) [46]. Therefore we investigated the impact of the *sec61-S353C* mutant on cell growth at various temperatures (37°C, 30°C, 25°C, 20°C) on YPD with and without tunicamycin. As positive controls we used the temperature- and cold-sensitive *sec61–3* mutant, and the cold-sensitive *sec61–32* mutant, both of which are import- and ERAD-defective [3, 4]. Optimal growth was observed at 30°C and 25°C for all strains, whereas at 37°C and 20°C growth was reduced (Fig. 3A). At 30°C in the absence of tunicamycin (Fig. 3A, 30°C, left) growth of all mutants was comparable to wildtype. With tunicamycin (Fig. 3A, right) growth was generally reduced for all strains, but only *sec61-32* displayed a tunicamycin-sensitivity in comparison to the wildtype (Fig. 3A, 30°C, right). At 37°C in the absence on tunicamycin growth of both *sec61-S353C* and *sec61–3* was reduced compared to wildtype (Fig. 3A, 37°C, left). The *sec61-S179P/S353C* double mutant, however, grew like wildtype at 37°C suggesting intramolecular suppression of the defect in *sec61-S353C* (Fig. 3A, 37°C, left). At this temperature growth of all strains was reduced in the presence of tunicamycin and indistinguishable from wildtype (Fig. 3A, 37°C, right). At 25°C growth of all mutants was comparable to wildtype both with and without tunicamycin, except for the cold sensitive *sec61–32* mutant, which was unable to grow with tunicamycin (Fig. 3A, 25°C). At 20°C the growth of *sec61–32* cells was reduced further compared to wildtype, and a slight cold-sensitivity of *sec61–3* was also detectable (Fig. 3A, 20°C). All other mutants grew like wildtype at this temperature (Fig. 3A, 20°C). Overall the *sec61-S353C* mutant displayed no tunicamycin-sensitivity, no cold-sensitivity, and a moderate temperature-sensitivity which was compensated in the *sec61-S179P/S353C* double mutant.

We next investigated whether the UPR was induced in our new *sec61* mutants using reporter plasmids which expressed ß-galactosidase from a promoter with or without a UPR element (UPRE) [46]. Transformants were grown on YPD without or with tunicamycin, cells were lysed and ß-galactosidase activity measured using a chromogenic substrate [38]. We used two strains, Δ*der1* and *sec61–3*, in which the UPR is constitutively induced as positive controls [4, 47]. In all strains ß-galactosidase activity was 1.5–2-fold higher when strains were grown in the presence of tunicamycin (Fig. 3B). The *SEC61* wildtype, the *sec61–302* mutant, and its derivatives all displayed similar ß-galactosidase activity both without and with tunicamycin (Fig. 3B). In contrast, in the positive control strains *(*Δ*der1*, *secc61–3*) we observed a 2- to 3-fold increase in ß-galactosidase activity compared to wildtype in the absence of tunicamycin which was further increased in the presence of the drug (Fig. 3B). In addition, we investigated UPR induction by analysis of *HAC1* mRNA splicing, but also found no induction by *sec61–302* or any of its derivatives (not shown). Our data demonstrate that the UPR is not induced in the *sec61-S353C* mutant suggesting no major accumulation of misfolded proteins in the ER of the mutant cells.

### Effect of the *sec61-S353C* mutation on ERAD in intact cells

**Fig 4:**
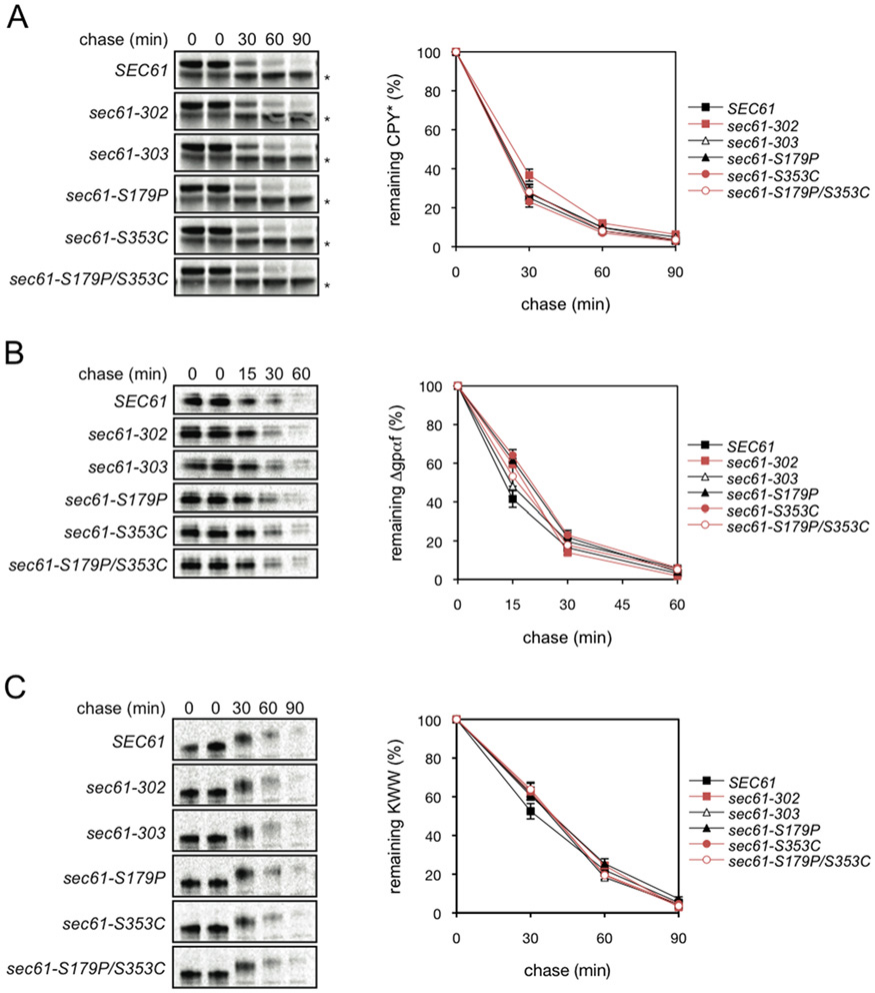
Effect of the *sec61-S353C* mutation on ERAD in intact cells. Mutants derived from *sec61–302* were investigated for ERAD of CPY*, Δgpαf and KWW. Wildtype and mutant cells were pulse-labeled with [^35^S]-methionine/cysteine, followed by chase incubations for the indicated periods of time. Cells were lysed, the respective proteins immunoprecipitated, separated by SDS-PAGE, and visualized by phosphorimaging. A: Proteins were pulse-labeled for 10 min. CPY* with an HA-Tag and endogenous CPY were precipitated with a polyclonal rabbit antibody against CPY. The position of mature endogenous CPY is indicated by an asterisk. B: Proteins were labelled for 5 min, and Δgpαf precipitated with a polyclonal rabbit antibody. C: KWW was precipitated with anti-HA antibody. Quantitations of CPY*, Δgpαf and KWW are shown in the graphs on the right. Mutants containing the S353C mutation are shown in red. Averaged data from 3 (A), or 2 (B, C) experiments are shown; bars indicate standard error.

As we had demonstrated previously that the 19S RP promotes exit of a soluble substrate from the ER [24], we next investigated the effects of *sec61–302* and its derivatives on ERAD of a Cdc48-dependent soluble substrate (CPY*), of a 19S RP-dependent soluble substrate (Δgpαf) and of a single-spanning transmembrane ERAD substrate with an ER-lumenal lesion (KWW) (Fig. 4). The Δgpαf mutant protein is a derivative of the yeast pro-α-factor mating pheromone precursor in which the N-glycosylation sites in the proregion were removed which causes misfolding [43]. Its export from the ER *in vitro* depends on the 19S RP both in yeast and mammalian microsomes [24, 25]. In the CPY* protein a point mutation (G255R) leads to misfolding and rapid export from the ER and degradation [47]. The export of CPY* is Cdc48-dependent [24, 48]. KWW is an artifical transmembrane ERAD substrate with a point mutation in its lumenal domain whose Cdc48- or 19S-dependence is not known [49]. Wildtype and mutant cells were pulse-labeled with [^35^S]-methionine/cysteine, followed by chase incubations for the indicated periods of time, lysis of cells, immunoprecipitation of ERAD substrates with appropriate antisera, separation of proteins by SDS-PAGE, and autoradiography. As shown in Fig. 4A and 4C, degradation of CPY* and KWW were barely affected in the *sec61* mutants we investigated: In agreement with published data CPY* had a t_1/2_ of about 21 min in wildtype cells and all mutants, except for *sec61–302* in which CPY* had a t_1/2_ of 24 min (Fig. 4A) [46]. KWW had a t_1/2_ of about 38 min in all *sec61* mutants investigated, but was degraded slightly faster in *SEC61* wildtype cells (t_1/2_ = 33 min; Fig. 4C). The 19S RP-dependently exported Δgpαf was the only substrate for which degradation kinetics were more variable in the *sec61* mutants: Whereas Δgpαf was exported from *SEC61* wildtype membranes and degraded with a t_1/2_ of 12 min (Fig. 4B, filled squares), turnover from *sec61–302* membranes (Fig. 4B, open squares), and *sec61-S353C* membranes (Fig. 4B, filled circles) was significantly slower with t_1/2_ of 19 min and 22 min, respectively. In the *sec61-S179P/S353C* double mutant Δgpαf had a t_1/2_ comparable to *sec61–302* (18 min, open circles, Fig. 4B). In summary, the *sec61-S353C* mutation, which reduces affinity of the Sec61 channel for the 19S RP, specifically affects turnover of an ERAD substrate whose export from the ER is 19S RP-dependent suggesting that the interaction may be functionally important for ERAD.

### The *sec61-S353C* mutation delays Δgpαf ERAD when proteasomes are limiting

**Fig 5:**
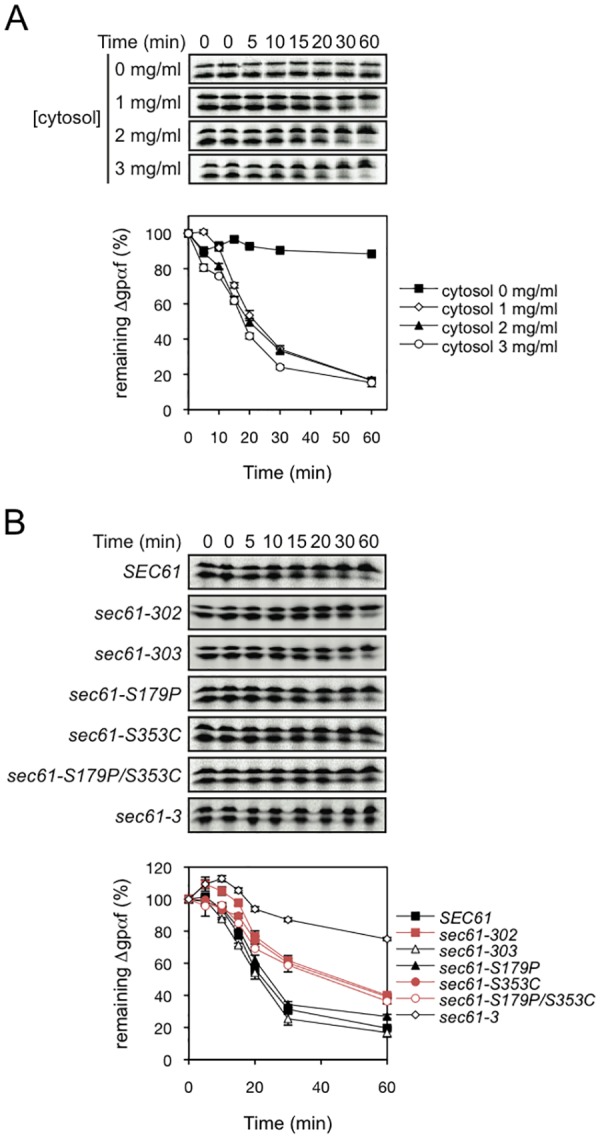
The *sec61-S353C* mutation delays ERAD when proteasomes are limiting. A: Limiting cytosol concentration was determined in an *in vitro* ERAD assay. *SEC61* wildtype microsomes were loaded with [^35^S]–methionine-labelled pΔgpαf, loaded membranes were washed, and incubated in the presence of ATP and the indicated concentrations of yeast cytosol for the indicated periods of time as described in [3]. Proteins were precipitated with TCA, separated by SDS-PAGE, and Δgpαf detected by autoradiography. Quantitation of Δgpαf (the lower band in each panel) is shown on the graph below. The upper band in each panel is signal sequence-containing pΔgpαf which aggregates on the cytoplasmic side of the membrane and cannot be washed off. B: Effects of *sec61* point mutations on ERAD were examined *in vitro* in presence of 1 mg/ml cytosol as above. At each time point Δgpαf was quantified and is shown in the graph below. Representative experiments are shown and the experiments were repeated twice.

As we had observed a modest effect of *sec61-S353C* on Δgpαf ERAD in intact cells, where proteasomes constitute about 1% of cellular protein, we next investigated ERAD of Δgpαf in a cell-free ERAD assay in which proteasomes can be made limiting [50, 24]. We have shown previously that export of Δgpαf is cytosol-dependent in this assay, and that the 19S RP is the only cytosolic factor required for Δgpαf export from the ER [3, 24]. Here we initially determined the cytosol concentration limiting for export, thus limiting the availability of 19S RP (Fig. 5A). *SEC61* wildtype yeast microsomes were loaded with [^35^S]-methionine- labeled pΔgpαf, and incubated in the presence of ATP and different concentrations of yeast cytosol for the indicated periods of time as described in [3]. Proteins were precipitated with TCA, separated by SDS-PAGE and Δgpαf detected by autoradiography. In the gels shown in Fig. 5A the lower band represents the ER-lumenal signal-cleaved Δgpαf which is exported and degraded in the presence of ATP and cytosol as a source of 19S RP (Fig. 5 A, upper panel). Cytosol concentrations of 3 mg/ml and 2 mg/ml appeared to be saturating, and export and degradation of Δgpαf proceeded with a t_1/2_ of 15 min at these concentrations, and without delay (Fig. 5A). In contrast, at 1 mg/ml cytosol initiation of export and degradation of Δgpαf was delayed by 10 min indicating a suboptimal concentration of 19S RP in the assay (Fig. 5 A, lower panel). Thus 1 mg/ml cytosol was used in the subsequent experiment as limiting cytosol concentration.

To investigate the effects of the *sec61-S353C* mutation on ER export when 19S RP are limiting, we then analyzed Δgpαf export from *sec61* mutant microsomes as described above in the presence of 1 mg/ml cytosol. Membranes from the export-defective *sec61–3* mutant served as control and showed a defect in ER export: only 30% of the substrate was degraded during the duration of the experiment (Fig. 5 B, 60 min) [3]. Membranes from *sec61–303* and *sec61-S179* yeast exported and degraded Δgpαf with wildtype kinetics (Fig. 5 B) and the t_1/2_ of Δgpαf with 1 mg/ml cytosol was about 20 min (Fig. 5B). In contrast, in mutants containing the S353C substitution in luminal loop 7 of Sec61p (*sec61–302*, *sec61-S353C*, *sec61-S179P/S353C*) ER export and degradation of Δgpαf were significantly delayed and its t_1/2_ increased to 45 min (Fig. 5B). Our data suggest that the 19S RP/Sec61 channel interaction is functionally important for Δgpαf export from the ER.

## Discussion

Proteasomes bind directly to the Sec61 channel via the 19S RP and in a cell-free assay can promote export of a soluble ERAD substrate from the ER [28, 24]. Here we show that the interaction of 19S RP with the Sec61 channel is important for export: we identify a new *sec61* mutant, *sec61-S353C*, that is import-competent (Fig. 1C), but has reduced affinity for the 19S RP (Fig. 2), and is export deficient for a 19S RP-dependent ERAD substrate (Figs 4 and 5).

We had previously identified two *sec61* mutants, *sec61–302* and *sec61–303*, which were both defective in cotranslational protein import into the ER, but only *sec61–302* had a proteasome 19S RP binding defect [29]. Of the four point mutations in *sec61–302* (Fig. 1A), two, D168G and F263L, were also present in *sec61–303* which was competent for proteasome binding to the Sec61 channel [29]. Here we therefore generated *sec61* mutants containing one or both of the remaining mutations in *sec61–302* (S179P and S353C; Fig. 1A) to determine which of these was responsible for the proteasome binding defect. When we analyzed the protein import competence into the ER of these *sec61* mutants we found that *sec61-S179P*, *sec61-S353C*, and the double mutant had no cotranslational or posttranslational import defects in contrast to the parental *sec61–302* mutant (Fig. 1C) suggesting that D168G and F263L were responsible for the cotranslational import defects in both *sec61–302* and in *sec61–303* (Fig. 1A). Our data further indicate that the amino acid substitutions S179P and S353C do not affect ribosome binding to or nascent chain insertion into the Sec61 channel.

When we analyzed the binding of 19S RP to mutant Sec61 channels derived from *sec61–302* to our surprise we found that the *sec61-S353C* substitution in ER-lumenal loop 7 was responsible for the reduced 19S RP binding to *sec61–302* channels (Fig. 2B). The *sec61-S179P* mutant displayed wildtype 19S RP interaction, whereas the *sec61-S179P/S353C* double mutant had an intermediate phenotype (Fig. 2B). We propose that the amino acid substitution in *sec61-S353C* alters the structure of L7 in the ER lumen, and that this altered L7 conformation is transmitted to the cytosolic face of the Sec61 channel, and can be detected by the proteasome 19S RP. This conformational change in the *sec61-S353C* mutant does not affect co- or posttranslational import into the ER (Fig. 1C), hence interactions of the mutant channel with ribosomes and the Sec63 complex must remain intact [7, 6]. Secondary structure prediction suggests an extension of L7 in *sec61-S353C* compared to the wildtype, concomitant with a reduction of the length of the α-helix in the loop [51, 52]. This extended L7 would push transmembrane helix 7 of Sec61p towards helix 2 and thus lead to stabilization of the lateral gate in the closed conformation on the lumenal side (Fig. 1A). Since the transmembrane domains of Sec61p form a tightly connected bundle, rigid extension of L7 on the lumenal side likely induces subtle shifts of the cytosolic ends of transmembrane domains 7, 8 and 2, and hence alters the cytoplasmic face of the channel [10, 11, 12]. In the *sec61-S179P/S353C* double mutant it appears that the S179P substitution was able to partially compensate for the defect in 19S RP binding to the mutant channel caused by S353C (Fig. 2B). Since proline increases the rigidity of protein structures, it may stabilize the cytoplasmic face of the Sec61 channel against the conformational change induced by S353C and thus preserve the 19S RP binding site [53].

If reduced binding of 19S RP to the *sec61-S353C* mutant channel had an effect on proteostasis in the ER, it should result in increased sensitivity to tunicamycin or elicit the UPR [16, 54]. This, however, was not the case (Fig. 3). The only indication of a defect in the *sec61-S353C* mutant was a modest temperature sensitivity at 37°C in comparison to the wildtype (Fig. 3A, top, left panel). It has been shown previously that amino acid substitutions in L7 affect Sec61 channel function at extreme temperatures: the *sec61–3* mutant (G341E) causes both temperature- and cold-sensitivity, whereas deletion of L7 causes cold-sensitivity only (Fig. 3A) [32, 55, 16]. We have also identified amino acid substitutions likely adaptive for Sec61 channel function in the cold in Sec61 L7 of polar fishes [13]. At 37°C the conformational change in Sec61p induced by the S353C substitution may be enhanced and lead to instability of the protein, and hence interfere with viability. Temperature-sensitivity of the *sec61-S353C* mutant was not exacerbated by tunicamycin suggesting that it was independent of ERAD. The *sec61-S179P/S353C* double mutant was not temperature-sensitive (Fig. 3A, top, left) confirming that the S179P substitution had a stabilizing effect which counteracted that of the S353C mutation.

When we investigated the effect of *sec61-S353C* on ERAD in intact cells with pulse-chase experiments, we found that the mutation moderately affected ERAD of Δgpαf, but not of CPY* or KWW (Fig. 4). Since export from the ER of Δgpαf is 19S RP-dependent whilst the primary driver of export of CPY* from the ER is Cdc48p, whereas the export motor for KWW is unknown, our data are consistent with 19S RP binding to the Sec61 channel being the limiting step for export of Δgpαf [24, 43, 49]. Subunits of the 19S RP and 20S CP are expressed stoichiometrically, and the total amount of proteasomes per yeast cell has been estimated to be between 15,000 and 30,000 which would be equivalent to 0.65–1.3 µM if proteasomes were distributed evenly throughout the cell [50]. Morphological examination suggests that at least 80% of proteasomes are located in the nucleus during exponential growth, which would reduce the cytosolic concentration to maximally 130–260 nM [50]. The K_D_ for proteasome binding to the Sec61 channel in microsomes is between 20 and 30 nm, hence in intact cells a reduction in affinity of the Sec61-S353C channel for the 19S RP by a factor of 2 or 3 would be compensated by the high concentration of proteasomes in the cytosol, and the effect on Δgpαf degradation should be modest which is what we observe (Figs. 2B and 4B) [28].

We have demonstrated previously that 19S RP is the only cytosolic factor required for ER export of Δgpαf *in vitro* [24]. The saturating cytosol concentration normally used in our reconstituted *in vitro* ERAD assay is 3 mg/ml [3, 43]. This is in the order of 10% of cytosolic protein concentration in the cell and hence provides 13–26 nM proteasomes which still is in the saturating range for wildtype Sec61 channels (Fig. 5A) [28]. Comparable to the effect in intact cells, 3 mg/ml cytosol were also partially able to compensate export deficiency in S353C mutant membranes (data not shown). A threefold dilution to 1 mg/ml cytosol, however, reduces the 19S RP concentration in the assay to a range that was suboptimal for 19S RP-driven export through the wildtype channel (Fig. 5A) and that allowed clear detection of the export defect in the S353C mutants with reduced 19S RP affinity (Fig. 5B).

ERAD of Δgpαf *in vitro* with *sec61-S179P/S353C* mutant membranes was delayed to the same degree as in the S353C mutant despite the fact that 19S RP binding was only modestly reduced (Fig. 5B, Fig. 2B). This might suggest that 19S RP binding to the channel does not only require a specific conformation of its cytoplasmic face, but also that the interaction, like ribosome binding to the Sec61 channel, induces a conformational change in the Sec61 channel that serves to prime export [56]. In this scenario whereas the destabilization of the cytoplasmic 19S RP binding site by S353C would be suppressed by S179P, the S353C mutation would still interfere with the priming step required for export and hence the double mutant would remain export deficient (Fig. 2B, 5B). In addition or alternatively, binding of lumenal factors required for triggering channel opening like chaperones could be affected by the S353C mutation which might explain the export deficiency of the *sec61-S179P/S353C* mutant despite its relatively efficient 19S RP binding (Fig. 5B, Fig. 2B).

Here we have identified and characterized the second export-specific *sec61* mutant, *sec61-S353C*. The first, *sec61-Y344H* was identified in a screen for mice prone to diabetes [14]. The homologous substitution in yeast, Y345H, causes a delay in ERAD of CPY* in intact yeast, but like the S353C mutant, does not induce tunicamycin sensitivity or the UPR [15, 16]. In contrast to *sec61-S353C*, the Y345H substitution had no effect on 19S RP binding, but a complete deletion of L7 resulted in enhanced affinity of 19S RP for the Sec61ΔL7 channels [16]. Modelling of the Sec61ΔL7 mutant channels suggested a partial opening of the lateral gate and crosslinking to Sss1p indicated that interactions between the two channel subunits were altered [16]. Taken together, our data from the previous paper and this work suggest that a conformational change hinging on L7 governs 19S RP interaction with the Sec61 channel which then results in export of specific ERAD substrates and their degradation in the cytosol.
